# Supplementary material for: International Delphi consensus on acute kidney injury: Foundations for AI-driven digital twin development in critical care nephrology
Source: PLoS One. 2026 Mar 25;21(3):e0344991. doi: 10.1371/journal.pone.0344991 (PMC13016343; doi:10.1371/journal.pone.0344991)
Supplement: S1 File — (PDF) [file pone.0344991.s001.pdf]

Supplemental File 1
Delphi Round 1 Questionnaire

Manuscript Title:
International Delphi Consensus on Acute Kidney Injury: Foundations for AI-Driven Digital Twin Development in Critical Care Nephrology

Instructions

Participants were asked to rate each statement using a 10-point Likert scale:

1 = Strongly disagree
10 = Strongly agree

Consensus was predefined as ≥75% of respondents rating a statement ≥8.

Section 1: Etiology and Pathophysiology

1. Comprehensive etiologic classification is essential for AI-based AKI modeling.
2. Hemodynamic parameters should be core variables in digital twin modeling.
3. Inflammatory biomarkers should be incorporated into AKI digital twin systems.
4. Tubular injury biomarkers provide essential mechanistic insight for digital modeling.
5. Multiorgan interaction modeling is required for accurate AKI simulation.

Section 2: Clinical and Laboratory Variables

6. Serum creatinine trends are essential but insufficient alone for modeling AKI.
7. Urine output dynamics must be incorporated into digital twin frameworks.
8. Acid–base status should be included in predictive modeling systems.
9. Lactate levels should be integrated into AKI digital twin modeling.
10. Vasopressor exposure should be included as a core modeling variable.

Section 3: Imaging and Histopathology

11. Imaging biomarkers should be incorporated when available.
12. Kidney biopsy findings can enhance AI-based phenotype classification in selected cases.

13. Histopathologic patterns may provide mechanistic insight into digital modeling systems.

Section 4: AI Infrastructure and Implementation

14. Real-time data integration is required for effective digital twins.
15. Continuous physiologic monitoring improves predictive modeling accuracy.
16. Interoperability with electronic health records is essential.
17. Model transparency and explainability are necessary.
18. Ethical safeguards must be integrated into AI-based frameworks.

Open-Ended Questions

19. What key variables are currently missing from AKI modeling approaches?
20. What are the primary barriers to implementing digital twin systems in critical care nephrology?
21. Additional comments or recommendations:
