# Supplementary material for: International Delphi consensus on acute kidney injury: Foundations for AI-driven digital twin development in critical care nephrology
Source: PLoS One. 2026 Mar 25;21(3):e0344991. doi: 10.1371/journal.pone.0344991 (PMC13016343; doi:10.1371/journal.pone.0344991)
Supplement: S2 File — (PDF) [file pone.0344991.s002.pdf]

Supplemental File 2
Delphi Round 2 Questionnaire

Manuscript Title:
International Delphi Consensus on Acute Kidney Injury: Foundations for AI-Driven Digital Twin Development in Critical Care Nephrology

Instructions

Participants rated each statement using the same 10-point Likert scale:

1 = Strongly disagree
10 = Strongly agree

Consensus threshold remained ≥75% rating ≥8.

Section 1: Revised Clinical and Modeling Variables

1. Dynamic creatinine trajectory provides more predictive value than absolute creatinine alone.
2. Time-weighted hypotension should be incorporated into AKI digital twin systems.
3. Integration of inflammatory biomarkers improves modeling precision.
4. Multiorgan dysfunction scoring systems should be integrated into digital twins.

Section 2: AI Governance and Deployment

5. Digital twin systems should provide real-time bedside risk prediction.
6. Predictive models must undergo external validation before clinical deployment.
7. Continuous recalibration of AI models is required.
8. Data governance frameworks are essential for implementation.

Final Consensus Statements

9. A standardized international AKI data dictionary is required.
10. Multicenter collaboration is necessary to train AI systems.

11. Explainable AI is mandatory in critical care deployment.
12. Ethical oversight committees should supervise digital twin implementation.

Final Comments

13. Please provide any additional recommendations regarding digital twin development in AKI.
