# Supplementary material for: International Delphi consensus on acute kidney injury: Foundations for AI-driven digital twin development in critical care nephrology
Source: PLoS One. 2026 Mar 25;21(3):e0344991. doi: 10.1371/journal.pone.0344991 (PMC13016343; doi:10.1371/journal.pone.0344991)
Supplement: Attachment — Submitted filename: Reviewers response.docx [file pone.0344991.s005.docx]

Manuscript ID: PONE-D-25-64532

Title: *International Delphi Consensus on Acute Kidney Injury: Foundations for AI-Driven Digital Twin Development in Critical Care Nephrology*

Dear Editor and Reviewers,

We sincerely thank the Academic Editor and both reviewers for their thoughtful and constructive evaluation of our manuscript. We greatly appreciate the time and expertise invested in reviewing our work.

Before addressing the reviewer comments, we confirm that we have ensured full compliance with PLOS ONE’s formatting and licensing requirements. Specifically:

- Documentation confirming licensing compliance for Figure 2 has been uploaded with this revision.
- All institutional and platform logos have been removed from the Supporting Information files to comply with CC BY 4.0 policies.
- Table 1 is now appropriately cited in the manuscript text.
- The manuscript has been formatted according to PLOS ONE guidelines.
- All major and minor revisions are clearly highlighted in red in the revised manuscript.

**Response to the Academic Editor**

In response to the Editor’s request to strengthen the discussion of the Delphi results, we have substantially revised and expanded the Discussion section. Specifically, we have:

- Provided deeper contextualization of the Delphi findings relative to international recommendations and routine ICU practices.
- Clarified the translational gap between biomarker endorsement and real-world ICU implementation.
- Expanded discussion of contrast use in AKI, desmopressin practices, and the influence of panel composition (including subspecialty distribution and geographic representation).
- Streamlined redundant sections related to the Delphi–DAG–digital twin framework to improve clarity and focus.

**Response to Reviewer #1**

We thank Reviewer #1 for the positive evaluation of the scientific rigor and conceptual importance of our work.

All minor comments have been addressed, including 1) correction of the incomplete sentence in the Introduction section, 2) clarification of the Delphi methodology by separating planned design from achieved results, 3) addition of methodological references supporting the consensus threshold, 4) definition of all abbreviations at first use, 5) clarification of biomarker terminology, 6) expanded reporting of KDIGO stage III findings, 7) definition of THAM, and 8) detailed description of the DAG construction process.

Figure 3 has been revised for clarity, and the figure legend has been updated to explicitly explain the framework and avoid confusion regarding round-specific arrows and biomarker categorization.

Table 1 has been expanded to provide detailed round-by-round results, including agreement percentages and consensus status.

In response to the major comments, we substantially revised the Discussion section to critically analyze the Delphi findings, compare them with international recommendations and routine ICU practice, discuss limitations related to panel composition and geographic predominance, explicitly address desmopressin use, and reduce redundancy in the discussion of the digital twin conceptual framework.

**Response to Reviewer #2**

We thank Reviewer #2 for the positive assessment of the manuscript’s scientific validity.

We corrected the incomplete sentence in the Introduction section, added appropriate methodological references, and clarified the total number of experts invited and participating in the Delphi process.

We believe these revisions have substantially strengthened the manuscript’s clarity, methodological transparency, and clinical contextualization. We are grateful to the reviewers for their insightful comments, which have significantly improved the rigor and presentation of our work.

Sincerely,
Kianoush B. Kashani, MD, MS
Corresponding Author
On behalf of all authors
